# Relative Contributions of Extracellular and Internalized Bacteria to Early Macrophage Proinflammatory Responses to Streptococcus pneumoniae

**DOI:** 10.1128/mBio.02144-19

**Published:** 2019-09-24

**Authors:** Jimstan Periselneris, Giuseppe Ercoli, Tracey Pollard, Suneeta Chimalapati, Emilie Camberlein, Gabriella Szylar, Catherine Hyams, Gillian Tomlinson, Fernanda C. Petersen, R. Andres Floto, Mahdad Noursadeghi, Jeremy S. Brown

**Affiliations:** a Centre for Inflammation and Tissue Repair, UCL Respiratory, Division of Medicine, University College Medical School, Rayne Institute, London, United Kingdom; b Division of Infection and Immunity, University College London, London, United Kingdom; c Molecular Immunity Unit, Department of Medicine, University of Cambridge, MRC Laboratory of Molecular Biology, Cambridge, United Kingdom; d Department of Oral Biology, Faculty of Dentistry, University of Oslo, Oslo, Norway; Albert Einstein College of Medicine; University of Mississippi Medical Center

**Keywords:** *Streptococcus pneumoniae*, capsule, TLR2, inflammation

## Abstract

Both intracellular immune sensing and extracellular innate immune sensing have been implicated in initiating macrophage proinflammatory cytokine responses to Streptococcus pneumoniae. The S. pneumoniae capsule, a major virulence determinant, prevents phagocytosis, and we hypothesized that this would reduce activation of host innate inflammatory responses by preventing activation of intracellular proinflammatory signaling pathways. We investigated this hypothesis in human monocyte-derived macrophages stimulated with encapsulated or isogenic unencapsulated mutant S. pneumoniae. Unexpectedly, despite strongly inhibiting bacterial internalization, the capsule resulted in enhanced inflammatory cytokine production by macrophages infected with S. pneumoniae. Experiments using purified capsule material and a Streptococcus mitis mutant expressing an S. pneumoniae serotype 4 capsule indicated these differences required whole bacteria and were not due to proinflammatory effects of the capsule itself. Transcriptional profiling demonstrated relatively few differences in macrophage gene expression profiles between infections with encapsulated S. pneumoniae and those with unencapsulated S. pneumoniae, largely limited to reduced expression of proinflammatory genes in response to unencapsulated bacteria, predicted to be due to reduced activation of the NF-κB family of transcription factors. Blocking S. pneumoniae internalization using cytochalasin D had minimal effects on the inflammatory response to S. pneumoniae. Experiments using murine macrophages indicated that the affected genes were dependent on Toll-like receptor 2 (TLR2) activation, although not through direct stimulation of TLR2 by capsule polysaccharide. Our data demonstrate that the early macrophage proinflammatory response to S. pneumoniae is mainly dependent on extracellular bacteria and reveal an unexpected proinflammatory effect of encapsulated S. pneumoniae that could contribute to disease pathogenesis.

## Abstract (precis)

Multiple extra- and intracellular innate immune receptors have been identified that recognize Streptococcus pneumoniae, but the relative contributions of intra- versus extracellular bacteria to the inflammatory response were unknown. We have shown that intracellular S. pneumoniae contributes surprisingly little to the inflammatory responses, with production of important proinflammatory cytokines largely dependent on extracellular bacteria. Furthermore, although we expected the S. pneumoniae polysaccharide capsule to block activation of the host immune system by reducing bacterial internalization and therefore activation of intracellular innate immune receptors, there was an increased inflammatory response to encapsulated compared to unencapsulated bacteria, which is likely to contribute to disease pathogenesis.

## INTRODUCTION

Streptococcus pneumoniae is an important bacterial cause of otitis media, pneumonia, septicemia, and meningitis, resulting in significant worldwide morbidity and mortality (1). S. pneumoniae causes a rapid and robust inflammatory response, including production of the key cytokines tumor necrosis factor (TNF), interleukin-1β (IL-1β), and IL-6, which are critical for protective immunity (2–13). However, this inflammatory response can also contribute to pathogenesis: for example, by causing pulmonary consolidation, acute respiratory distress syndrome (ARDS), and septic shock and, in patients with meningitis, chronic neurological damage (14–16). Multiple mechanisms have been described that contribute to the inflammatory response to S. pneumoniae, including both intracellular and extracellular pathogen recognition receptors (PRRs). Extracellular PRRs activated by S. pneumoniae include Toll-like receptor 2 (TLR2 [by bacterial lipoproteins]) (17), TLR4 (by pneumolysin) (18), and the scavenger receptor MARCO (19). More recently, there has been considerable interest in activation of intracellular PRRs by S. pneumoniae. These include activation of the inflammasome by the pore-forming toxin pneumolysin (20), nucleotide-binding oligomerization domain (NOD) receptors by peptidoglycan (21, 22), and nucleic acid receptors such as cGAS (23) and RIG-I-like receptors (RLRs) (24). In addition, TLRs are also located in phagosome membranes, and can be activated by phagocytosed bacteria (25–27). A similar range of PRRs are involved in the inflammatory response to other extracellular bacterial pathogens; the macrophage-dependent inflammatory response to S. aureus has been shown to be dependent on both extracellular and internalized bacteria (25). However, the relative contributions to the macrophage inflammatory response of activation of intracellular PRRs by internalized S. pneumoniae versus activation of cell surface PRRs by extracellular bacteria have not been defined.

The most important S. pneumoniae virulence factor is the capsule, a polysaccharide layer that surrounds the bacterium and profoundly inhibits phagocytosis as well as preventing bacterial entrapment by respiratory mucus and killing by neutrophil extracellular traps (28–31). As unencapuslated S. pneumoniae strains are much more susceptible to phagocytosis than otherwise isogenic encapsulated strains (29, 32), comparing phagocyte inflammatory responses to unencapsulated and encapsulated S. pneumoniae may identify the relative importance of intracellular signaling pathways for inflammatory responses. In contrast to cell wall and membrane components, previously published data suggest that purified capsular polysaccharide does not stimulate an inflammatory response. Purified capsular material only induces low levels of inflammation in a rabbit meningitis model (33), in human whole blood (34), and in RAW murine macrophages (35). These weak responses may be caused by residual contamination of capsular material with proinflammatory cell wall and membrane components (36). As the capsule surrounds the bacterium and blocks phagocytosis, the capsule could also affect inflammation by blocking interaction of cell surface TLRs to S. pneumoniae ligands. Hence the capsule could potentially alter inflammatory responses to S. pneumoniae by reducing activation of both intracellular and cell surface PRRs. These questions have not previously been addressed despite the importance of both the capsule and inflammatory responses to S. pneumoniae disease pathogenesis.

Using *in vitro* infection of monocyte-derived macrophages (MDMs) with encapsulated and unencapsulated bacteria, supported by data from a mouse pneumonia model, we have investigated the relative contributions of macrophage cell surface and intracellular PRRs to the macrophage inflammatory response to S. pneumoniae. We hypothesized that intracellular PRRs would make a significant contribution to the inflammatory response to S. pneumoniae, such that the capsule would contribute to immune evasion by reducing the inflammatory response as a result of diminished bacterial phagocytosis.

## RESULTS

### Loss of the S. pneumoniae capsule increases internalization by macrophages.

**FIG 1:**
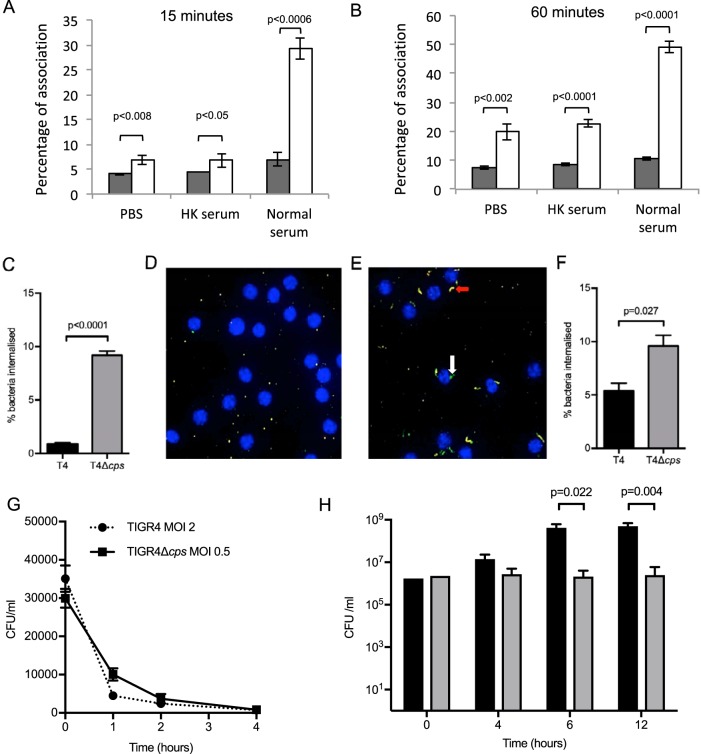
(A and B) RAW macrophages were incubated with FAM-SE-labeled bacteria preincubated in PBS, heat-inactivated (HK) serum, or 10% pooled human serum. Cells were washed at 2 time points and then lifted and analyzed by flow cytometry to assess association. The results of 4 experiments are displayed as means ± standard errors of the means (SEM) at (A) 15 min or (B) 60 min, with TIGR4 data shown as gray bars and TIGR4 Δ*cps* data as white bars, as analyzed by *t* test. (C) MDMs incubated with bacteria at an MOI of 10 had an antibiotic protection assay performed after 2 h, and data were analyzed by *t* test, with means ± SEM from 3 experiments displayed. WT TIGR4 data are shown as black bars and TIGR4 Δ*cps* data as gray bars. (D to F) High-throughput imaging of fluorescent TIGR4 and TIGR4 Δ*cps* labeled with green fluorophore and incubated for 2 h with MDMs (nuclei in blue), with external bacteria labeled with anti-human IgG (labeled red) appearing yellow (red arrow) due to the interaction of red and green fluorophores, whereas internalized bacteria remain green (white arrow). Sample images of MDMs incubated with TIGR4 and TIGR4 Δ*cps* are shown in panels E and F, respectively. (G) Numbers of external and internal bacteria were counted, and data are displayed as means ± SEM from 3 experiments, with analysis by unpaired *t* test. WT TIGR4 data are shown as black bars and TIGR4 Δ*cps* data as gray bars. (G) Once TIGR4 and TIGR4 Δ*cps* were internalized, with inocula adjusted such that internalized bacteria at time zero were similar, numbers of surviving internal bacteria were measured by antibiotic protection assay using THP-1 cells. Results are displayed as means ± SEM from 5 experiments at each time point, as analyzed by unpaired *t* test. WT TIGR4 data are displayed as squares joined by a solid line and TIGR4 Δ*cps* data as circles joined by a dotted line. (H) Bacteria were incubated with MDMs at an MOI of 10, and MDM supernatant was plated at specified time points, displayed as the median and range from 6 experiments, with WT TIGR4 data shown as black bars and TIGR4 Δ*cps* data as gray bars, with counts analyzed by the Mann-Whitney U test.

Previously we demonstrated that the capsule inhibits neutrophil phagocytosis of S. pneumoniae (29). Flow cytometry, microscopy, and antibiotic protection assays were used to confirm that loss of the capsule also increases S. pneumoniae phagocytosis by macrophages. In flow cytometry assays, the unencapsulated TIGR4 Δ*cps* bacteria were more rapidly associated with RAW macrophages than wild-type (WT) TIGR4 bacteria (Fig. 1A and B). Differences were evident after 15 min of incubation and were present whether bacteria were unopsonized or opsonized with complement-inactivated serum or normal human serum. Antibiotic protection assays confirmed that in 5% human serum, the percentage of adherent bacteria internalized by human MDMs after 2 h was nearly 10-fold greater for the TIGR4 Δ*cps* strain than that of WT TIGR4 (*P* < 0.0001) (Fig. 1C). This was supported by microscopy, which showed a 2-fold increase in uptake of unencapsulated bacteria (*P* = 0.042) (Fig. 1D to F) at 2 h. However, once internalized, there was no significant difference in the intracellular survival of TIGR4 and TIGR4 Δ*cps* bacteria when measured by antibiotic protection assays using cells of the human macrophage cell line THP-1 (Fig. 1G). Comparative intracellular survival experiments were not performed with MDMs as inherent interdonor variability in bacterial uptake prevents identification of bacterial numbers that consistently lead to the internalization of similar numbers of TIGR4 and TIGR4 Δ*cps* bacteria. The increase in macrophage phagocytosis allowed MDMs to control TIGR4 Δ*cps* bacterial numbers, whereas after 6 h, significantly more TIGR4 CFU were recovered from culture supernatants (Fig. 1H). Overall, these data demonstrate that incubation of unencapsulated S. pneumoniae with MDMs results in substantial increases in the numbers of intracellular bacteria compared to incubation with encapsulated bacteria, but without significantly affecting the dynamics of intracellular killing.

### Comparison of the MDM transcriptional responses to encapsulated and unencapsulated S. pneumoniae.

**FIG 2:**
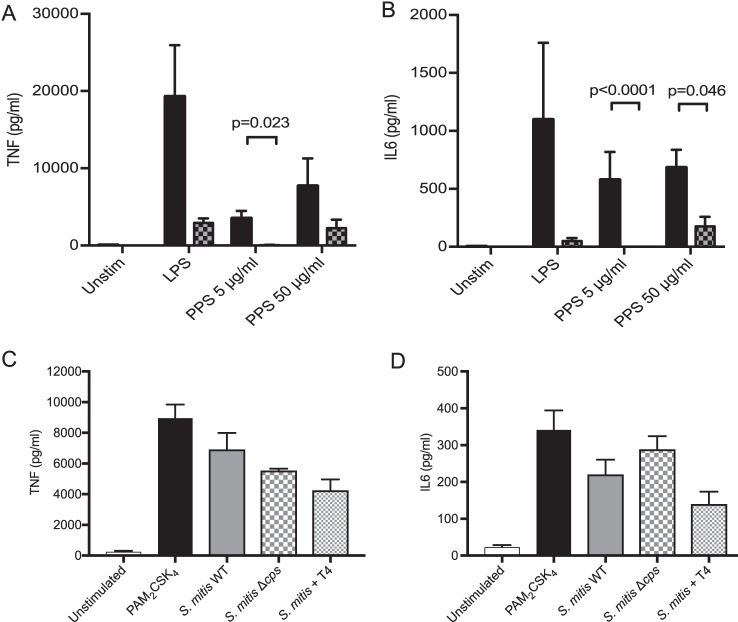
Direct effects of capsule on inflammatory responses. (A and B) MDMs were incubated with PPS (solid bars) with or without 30 μg/ml polymyxin B (PMxB) (checked bars), and TNF (A) and IL-6 (B) were measured. LPS at 100 ng/ml was used as a positive control, with and without addition of PMxB, and both sets of experiments are represented as means ± SEM from 3 experiments. Analysis was by 2-way ANOVA and Tukey’s multiple-comparison test. (C and D) MDMs were incubated with the S. mitis WT, S. mitis Δ*cps* mutant, or an S. mitis strain expressing the S. pneumoniae serotype 4 capsule at an MOI of 10. PAM_2_CSK_4_ at 100 ng/ml (Cell Signaling) was used as a positive control. TNF (C) and IL-6 (D) supernatant concentrations were measured after 6 h and are shown as means ± SEM from three separate experiments. The data were analyzed using 1-way ANOVA and Bonferroni multiple-comparison test.

Although purified serotype 4 polysaccharide induced MDM inflammatory cytokine secretion, this was largely abrogated by neutralization of contaminating lipopolysaccharide (LPS) using polymyxin B (Fig. 2A and B). To assess whether the capsule directly influences macrophage inflammatory response to external bacteria, MDMs were incubated with a Streptococcus mitis mutant strain expressing the S. pneumoniae serotype 4 capsule (37). Expression of the serotype 4 capsule by S. mitis did not enhance the MDM inflammatory response compared to the wild type or an unencapsulated mutant strain (Fig. 2C and D). These data show the capsule had limited direct effects on MDM inflammatory responses, and therefore we could exploit the increased phagocytosis of unencapsulated S. pneumoniae to compare the effects of internalized versus extracellular bacteria on the MDM response to S. pneumoniae.

**FIG 3:**
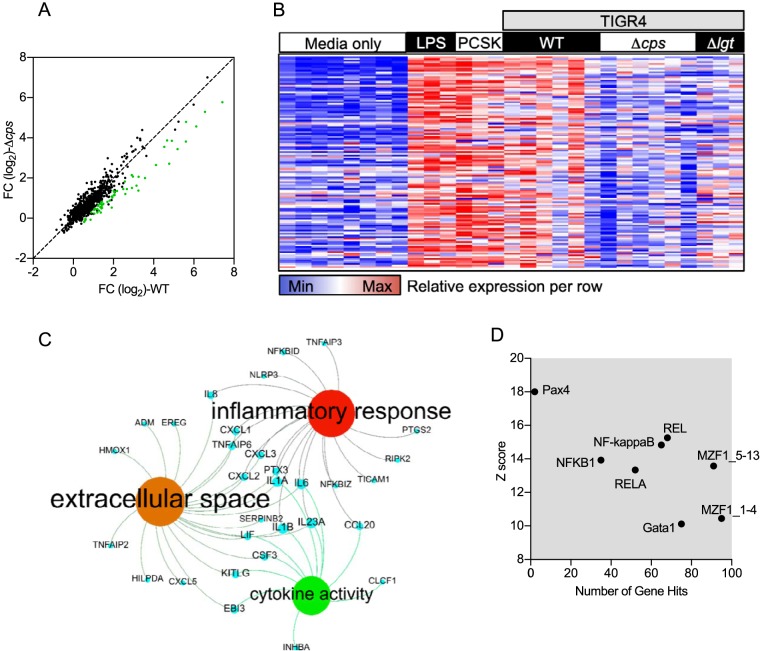
Whole-genome transcriptomic analysis was undertaken of MDMs incubated with TIGR4, TIGR4 Δ*cps*, or TIGR4 Δ*lgt* at an MOI of 10 or controls with medium only, PAM_2_CSK_4_ at 100 ng/ml (Cell Signaling), or LPS at 100 ng/ml (Cell Signaling), all at 4 h. Some cells were preincubated with 10 μM cytochalasin D for 30 min prior to the addition of bacteria. (A) Correlation of fold change (FC) in expression of genes in MDMs stimulated with TIGR4 or TIGR4 Δ*cps*. Here, each point represents a gene, with the genes attenuated due to TIGR4 Δ*cps* in comparison to those of WT TIGR4 represented in green. (B) All the genes expressed at a significantly higher level (*P* < 0.05, FC of >1.5) by WT TIGR4-stimulated MDMs in comparison to TIGR4 Δ*cps-*stimulated MDMs, as calculated by *t* test. Each column represents an individual experiment, with 6 donors each for unstimulated and the TIGR4- and TIGR4 Δ*cps*-stimulated groups and 3 each for all other conditions. (C) Network diagram illustrating the most significantly enriched gene ontologies from panel A, where blue nodes are genes, red nodes are biological processes, green nodes are molecular functions, and amber nodes are the cellular compartment. (D) Transcription factor binding site analysis for the transcriptional responses attenuated by TIGR4 Δ*cps*, showing transcription factor binding sites that are significantly enriched in the responses attenuated in the absence of capsule. This is plotted as the number of genes that each transcription factor is predicted to be activated by against the Z score, a statistical measure of degree of overrepresentation over background.

Microarrays were used to compare genome-wide transcriptional responses to MDM infection with either the TIGR4 or TIGR4 Δ*cps* strain (Fig. 3A). Overall, 294 genes showed increased expression in response to infection with either S. pneumoniae strain (Fig. 3A). Of these, a subset of 100 genes were upregulated in response to TIGR4 alone, 104 genes were upregulated in response to the TIGR4 Δ*cps* strain alone, and 194 genes were upregulated in response to both strains. As previously reported by us (17), the MDM genes that showed the greatest degree of upregulation in response to TIGR4 included those coding for the proinflammatory cytokines TNF, IL-23A, IL-6, IL-1β, and IL-8, along with a range of chemokines, and closely mirrored the MDM response to the TLR2 agonist PAM_2_CSK_4_ (Fig. 3B). Unexpectedly, the upregulation of many of these genes was substantially lower when MDMs were infected with the TIGR4 Δ*cps* strain. Differences in MDM transcripts upregulated after S. pneumoniae infection between the TIGR4 and TIGR4 Δ*cps* strains showed a partial attenuation in the proinflammatory subset of responses. This mirrored the attenuated response to the TIGR4 Δ*lgt* strain that lacks lipoproteins required for activation of TLR2 (17). Bioinformatic analysis of this subset by gene ontology showed that these were enriched for secreted inflammatory cytokines (Fig. 3C). Transcription factor binding site analysis inferred that these genes were primarily under the control of the NF-κB family (Fig. 3D). Hence, despite resulting in enhanced phagocytosis and internalization by MDMs, loss of the capsule specifically results in reduced proinflammatory MDM transcriptional responses to infection with S. pneumoniae.

### Encapsulated bacteria stimulate stronger MDM inflammatory cytokine responses.

**FIG 4:**
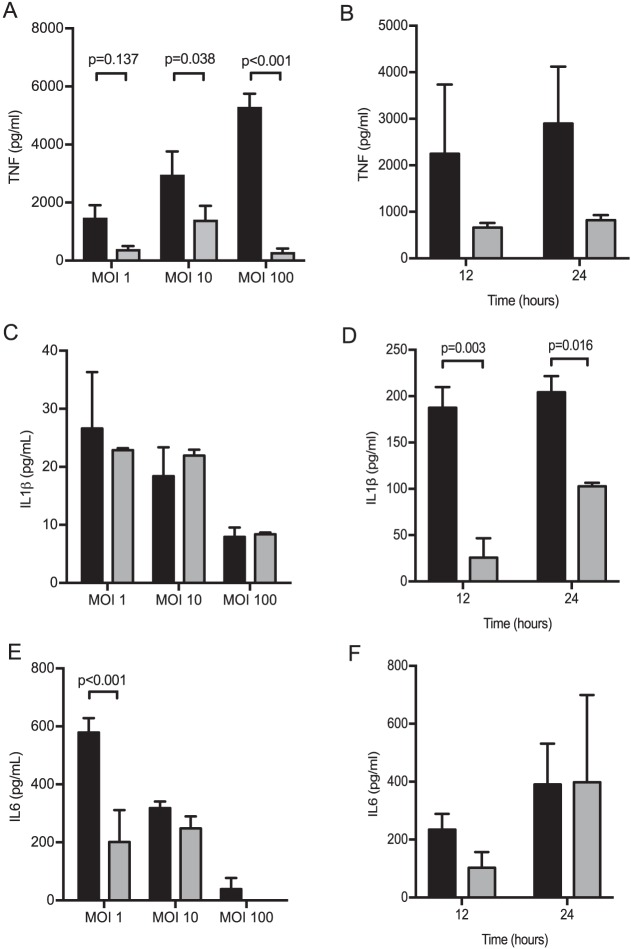
MDMs were incubated with TIGR4 or TIGR4 Δ*cps* for 6 h at various MOI, and supernatant was analyzed for (A) TNF, (C) IL-1β, and (E) IL-6, with TIGR4 data displayed as black bars and TIGR4 Δ*cps* data as gray bars. The data are displayed as means ± SEM from at least 3 separate experiments, as analyzed by unpaired *t* test. MDMs were incubated with TIGR4 or TIGR4 Δ*cps* at an MOI of 10, and supernatant was analyzed at various time points for (B) TNF, (D) IL-1β, and (F) IL-6. TIGR4 data are shown as black bars and TIGR4 Δ*cps* data as gray bars, and the data are displayed as means ± SEM from 3 experiments, as analyzed by unpaired *t* test.

To validate the changes to transcriptional responses to the TIGR4 Δ*cps* strain at the protein level, TNF, IL-1β, and IL-6 cytokine levels were measured in cell culture supernatants of MDMs incubated with the TIGR4 or the TIGR4 Δ*cps* strain. After 6 h, MDMs showed a dose-dependent increase in TNF responses to TIGR4 but not to TIGR4 Δ*cps*, with markedly higher levels of TNF in response to TIGR4 compared to TIGR4 Δ*cps* at multiplicities of infection (MOI) of greater than 10 (Fig. 4A). The TNF response to an MOI of 100 for the TIGR4 Δ*cps* strain was lower than that to an MOI of 10 or 1 for the TIGR4 strain. Nonsignificant differences in TNF levels persisted between TIGR4 and TIGR4 Δ*cps* infected MDMs 12 and 24 h after infection (Fig. 4B). After 6 h, there was little IL-1β release from MDMs at multiple MOI, but by 12 h, MDMs showed increased IL-1β production in response to the TIGR4 strain compared to TIGR4 Δ*cps* (Fig. 4C and D). Release of IL-6 decreased with increasing MOI for both strains; TIGR4 induced more IL-6 than TIGR4 Δ*cps* only at an MOI of 1, with no significant differences between the strains with higher MOIs and later time points (Fig. 4E and F). Overall, these data complement the transcriptional response results by demonstrating that the encapsulated TIGR4 strain stimulated a stronger MDM inflammatory cytokine response than the unencapsulated TIGR4 Δ*cps* strain, with major differences in production of TNF and some differences in IL-6 and IL-1β.

### The TIGR4 strain induces stronger early TNF responses in a mouse model of pneumonia.

**FIG 5:**
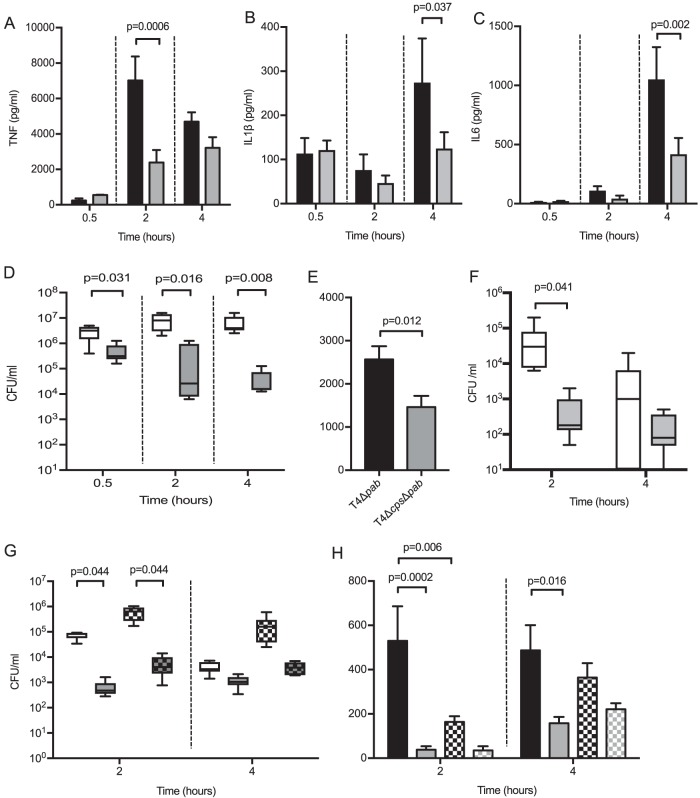
(A to D) Six-week-old female CD1 mice were infected with 5 × 10^6^ CFU of either TIGR4 or TIGR4 Δ*cps*, with 6 mice in each group. At various time points, mice were sacrificed, and BALF was retrieved and analyzed for (A) TNF, (B) IL-1β, and (C) IL-6. The results were analyzed by unpaired *t* test. (D) Bacterial CFU in BALF was measured at the same time points, as analyzed by Mann-Whitney U test. (E and F) Six-week-old female CD1 mice were infected with 5 × 10^6^ CFU of either TIGR4 Δ*pab* or TIGR4 Δ*cps* Δ*pab*, with 6 mice in each group, and after 2 h, mice were sacrificed and BALF was retrieved and analyzed for (E) TNF levels, as analyzed by unpaired *t* test, and (F) bacterial CFU in BALF was measured at the same time points, as analyzed by Mann-Whitney U test. (G and H) Six-week-old female CD1 mice were given intranasal liposomes or liposomal clodronate 72 h prior to infection with 5 × 10^5^ CFU of either TIGR4 or TIGR4 Δ*cps*, with 6 mice in each group. After 2 and 4 h, mice were sacrificed and BALF was retrieved for (G) bacterial CFU, as analyzed by Kruskal-Wallis test with Dunn’s multiple-comparison test and (H) TNF levels, as analyzed by 1-way ANOVA and Tukey’s multiple-comparison test. Black bars represent TIGR4 data and gray bars TIGR4 Δ*cps* data, with data from clodronate-treated mice shown as checked bars (as seen in panels G and H). For panels D and G, white boxes and whisker plots represent TIGR4 and gray ones TIGR4 Δ*cps*, with data from clodronate-treated mice in checked bars. In panel F, TIGR4 Δ*pab* data are displayed as white whisker plots, with TIGR4 Δ*cps* Δ*pab* data as gray plots.

A murine model of pneumonia (17, 32) was used to assess whether the differences seen in cell culture in TNF responses to S. pneumoniae reflected differences during disease. After high-dose infection (5 × 10^6^ CFU per mouse), bronchoalveolar lavage fluid (BALF) TNF levels recovered at 2 h and IL-1β and IL-6 at 4 h were higher in response to the TIGR4 strain compared to infection with the TIGR4 Δ*cps* strain (Fig. 5A to C). However, as previously described (32), the capsule played an important role in bacterial clearance, with markedly reduced levels of TIGR4 Δ*cps* CFU recovered in BALF (Fig. 5D). These differences in bacterial CFU in BALF confound interpretation of differences in cytokine responses. To partially account for this, experiments were repeated using the nonreplicating Δ*pabB* mutant TIGR4 and TIGR4 Δ*cps* strains to ensure BALF TNF responses reflect differences to the initial bacterial inoculum. Using Δ*pabB* strains, there was still an increased BALF TNF response to the encapsulated TIGR4 strain (Fig. 5E and F). To confirm that BALF TNF responses reflected alveolar macrophage interactions with S. pneumoniae and to try and reduce CFU differences between the strains, low-dose infection (5 × 10^5^ CFU) experiments were performed in mice treated with intranasal liposomal clodronate 72 h prior to infection, which depletes approximately 85% of alveolar macrophages (32). Although macrophage depletion resulted in higher CFU in BALF (Fig. 5G), both 2- and 4-h BALF TNF levels were markedly reduced in response to the TIGR4 strain (Fig. 5H). These data confirm that in a murine pneumonia model, there is an increased early TNF response to infection with encapsulated S. pneumoniae compared to unencapsulated bacteria, which is largely dependent on alveolar macrophages.

### MDM TNF responses to S. pneumoniae are largely dependent on extracellular bacteria.

**FIG 6:**
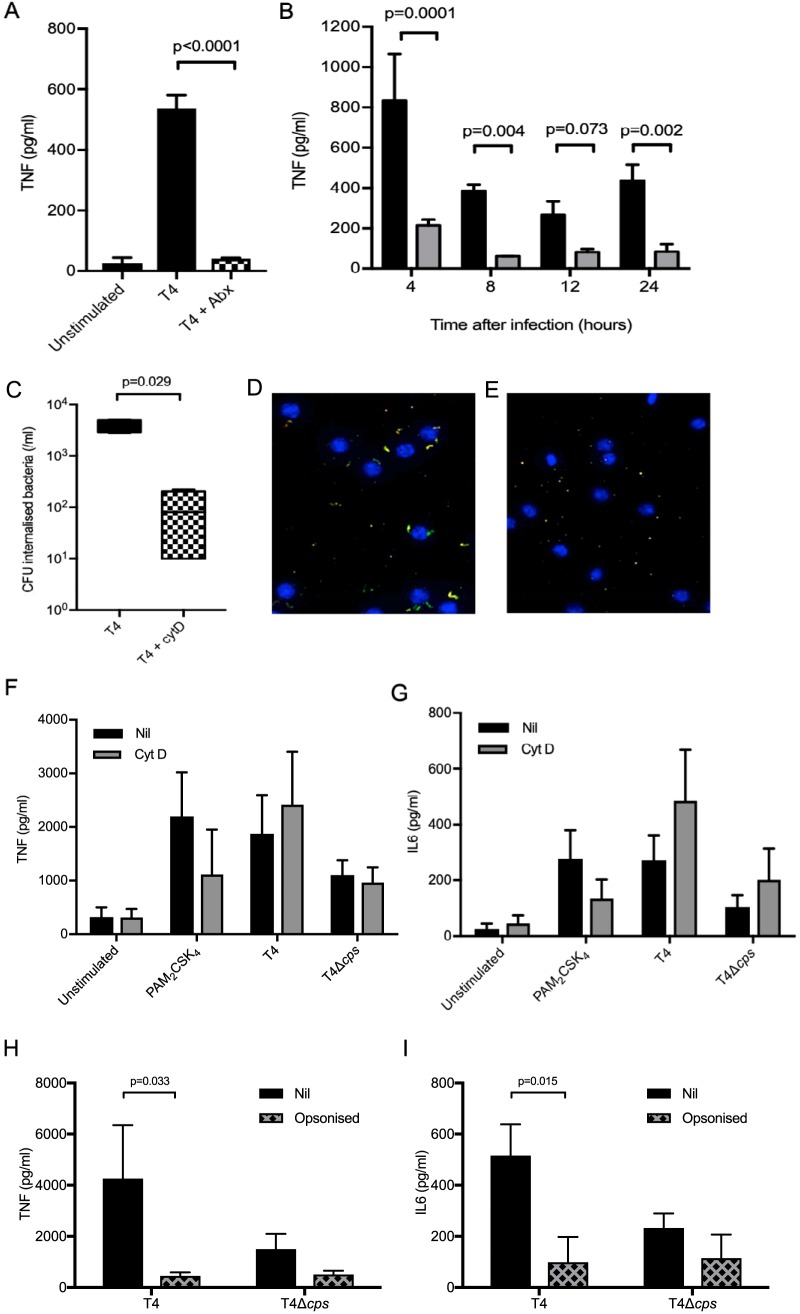
(A) MDMs were incubated with bacteria for 30 min at an MOI of 10, then medium or gentamicin was added, and supernatant was analyzed after 4 h for TNF levels. The TIGR4 response is displayed as a black bar, with the antibiotic-supplemented data as a checked bar, with means ± SEM from 4 experiments shown, as analyzed by unpaired *t* test. (B) MDMs were incubated with bacteria for 4 h at an MOI of 10, then medium or gentamicin was added, and supernatant was analyzed for TNF levels at subsequent time points. WT TIGR4 data are shown as black bars and TIGR4 Δ*cps* data as gray bars, displayed as means ± SEM from 3 experiments, as analyzed by *t* test. (C) MDMs were incubated with 10 μM cytochalasin D or medium for 30 min and then incubated with TIGR4 or TIGR4 Δ*cps*, and the antibiotic protection assay was used to determine numbers of internalized bacteria. Box and whisker plots of median and range of experiments are shown with TIGR4 data as a black box and cytochalasin D-treated cell data as a checked box, as analyzed by Mann-Whitney U test. (D and E) MDMs were incubated with fluorescently labeled TIGR4 (in green) at an MOI of 10 after preincubation with medium only or 10 μM cytochalasin D and imaged on a high-throughput microscope after 2 h, with nuclei in blue and with external bacteria in yellow. (D) Representative image of TIGR4 only and (E) TIGR4 plus cytochalasin D. (F and G) MDMs were incubated with controls or bacteria at an MOI of 10 for 6 h after preincubation with medium or 10 μM cytochalasin D, and (F) TNF and (G) IL-6 were measured in supernatant. Data are presented as mean ± SEM from at least 3 experiments, as analyzed by unpaired *t* test. (H and I) Bacteria were incubated with pooled human serum for 30 min, before incubation with MDM at an MOI of 10 for 6 h, and then supernatant (H) TNF and (I) IL-6 were measured. The data are presented as means ± SEM from at least 3 experiments, as analyzed by 2-way ANOVA and Tukey’s multiple-comparison test.

The above data suggest that MDM TNF responses to S. pneumoniae are not dependent on internalized bacteria. To confirm this and identify how long MDMs need to be exposed to S. pneumoniae to stimulate an effective inflammatory response, MDMs were incubated with S. pneumoniae TIGR4 and then treated with gentamicin to kill extracellular bacteria after phagocytosis of a significant number of bacteria had already occurred. There was no significant 4-h TNF response by MDMs exposed to extracellular TIGR4 for only 30 min, whereas 4 h of exposure stimulated a significant TNF response that persisted for 24 h and was consistently increased in response to the TIGR4 strain compared to the TIGR4 Δ*cps* strain (Fig. 6A and B). In contrast, 4 h of exposure of MDMs to TIGR4 resulted only in very low levels of supernatant IL-1β and IL-6, possibly because production of these cytokines in general needs a more prolonged stimulus (see Fig. S1A and B in the supplemental material). To further investigate the relationship between intra- and extracellular bacteria and the macrophage inflammatory response, MDM infection experiments were repeated with the addition of cytochalasin to block phagocytosis. Cytochalasin at a concentration of 10 μM inhibited S. pneumoniae phagocytosis by MDMs (Fig. 6C to E). Despite this, cytochalasin did not reduce MDM supernatant TNF, IL-6 (Fig. 6F and G), and IL-1β (not shown) responses to infection with either the TIGR4 or the TIGR4 Δ*cps* strain, with most responses actually showing non-statistically significant increases. Opsonization of S. pneumoniae with human serum increased phagocytosis and internalization of bacteria (29) but reduced supernatant levels of TNF and IL-1β and abrogated differences between the wild-type and unencapsulated strains (Fig. 6H and I). These data suggest that the MDM inflammatory response to S. pneumoniae is largely driven by extracellular rather than intracellular bacteria.

10.1128/mBio.02144-19.1 FIG S1 MDMs were incubated with bacteria for 4 h at an MOI of 10, then medium or gentamicin was added, and supernatant was analyzed for (A) TNF or (B) IL-6 levels at subsequent time points. WT TIGR4 data are shown as gray bars and TIGR4 Δ*cps* data as gray bars, displayed as means ± SEM from 3 experiments, as analyzed by *t* test. (C) Bacteria were incubated at varied concentrations with horse blood and supernatant, and the degree of hemolysis was measured by absorbance at 540 nm. A representative example from 4 experiments is shown. (D and E) MDMs were incubated with WT TIGR4 (black bars), TIGR4 Δ*ply*, or TIGR4 Δ*ply* Δ*cps* at an MOI of 10 for 6 h, and supernatant was analyzed for (D) TNF or (E) IL-6. The means ± SEM from 3 experiments are displayed, as analyzed by 1-way ANOVA and Tukey’s multiple-comparison test. Download FIG S1, TIF file, 2.8 MB. Copyright © 2019 Periselneris et al. 2019 Periselneris et al. This content is distributed under the terms of the Creative Commons Attribution 4.0 International license.

**FIG 7:**
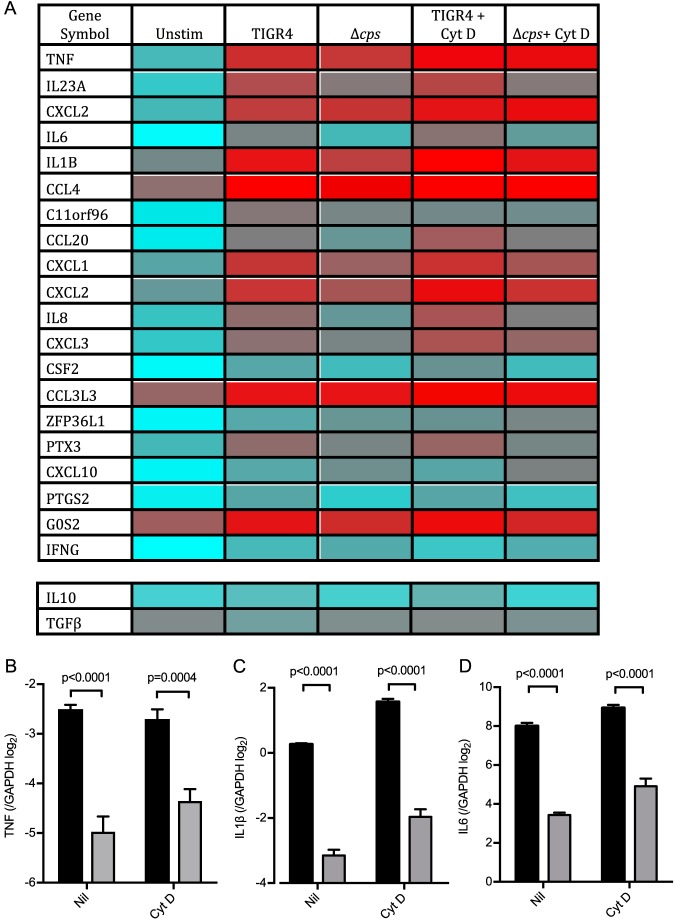
Mean gene expression of MDMs incubated with WT TIGR4 or the Δ*cps* mutant (6 donors each), with the least-expressed genes in blue and the most-expressed genes in red. They are displayed in gene order by most upregulated genes at the top, with TIGR4 stimulated compared to unstimulated (Unstim). The expression of two important anti-inflammatory cytokines is also displayed in two separate rows. In the relevant experiments, MDMs were preincubated with 10 μM cytochalasin D for 30 min, which was washed off, before the addition of bacteria at an MOI of 10. (B to D) MDMs were incubated with TIGR4 (black bars) or TIGR4 Δ*cps* (gray bars) at an MOI of 10 for 4 h, and lysates were analyzed by qPCR for (B) TNF, (C) IL-1β, and (D) IL-6. The mean ± SEM Δ*C_T_* from 4 experiments is displayed, as analyzed by unpaired *t* test.

Transcriptional analysis was used to assess in detail the effects of blocking phagocytosis on MDM response to encapsulated and unencapsulated S. pneumoniae. Incubating MDMs with cytochalasin D had relatively minor effects on the overall MDM transcriptional response to S. pneumoniae, although overall there was a general increase in inflammatory gene expression with preservation of the differences between the TIGR4 and TIGR4 Δ*cps* strains (Fig. 7A). There was little upregulation and few major differences elicited between strains in the expression of the anti-inflammatory cytokine genes coding for IL-10 or transforming growth factor β (TGF-β) (Fig. 7A). Quantitative PCR (qPCR) indicated small increases in expression of the IL-1β and IL-6 genes but not TNF transcripts after MDMs were treated with cytochalasin D, but with preservation of the large differences between the TIGR4 and TIGR4 Δ*cps* strains (Fig. 7B to D). Overall, these data confirm that the MDM proinflammatory cytokine responses to S. pneumoniae are largely dependent on extracellular bacteria.

### MDM responses to other unencapsulated strains and role of TLR2 activation.

**FIG 8:**
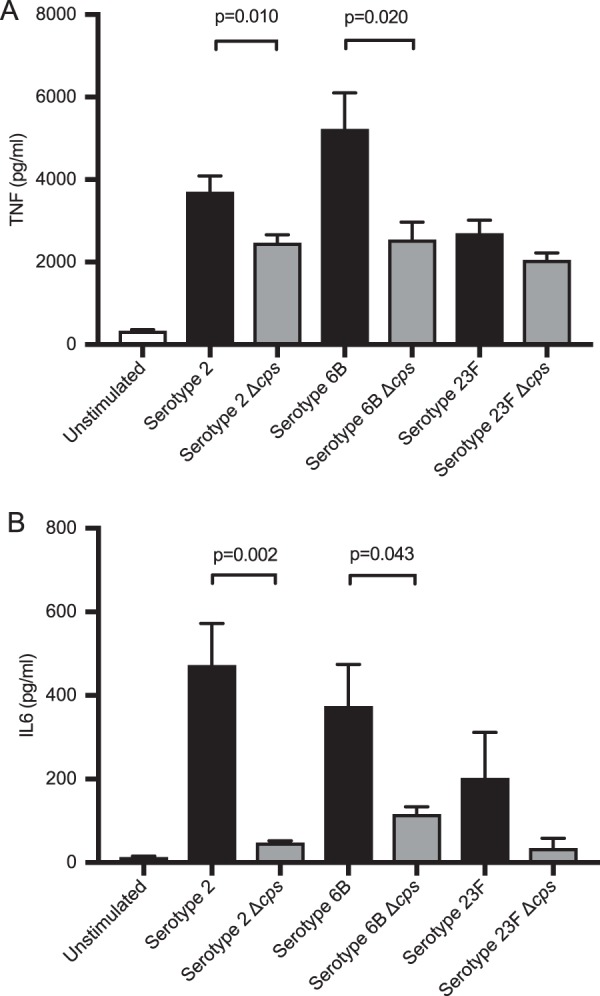
(A and B) MDMs were incubated with wild-type bacteria (serotypes 2, 6B, and 23F), with WT data shown as black bars and isogenic capsule-deficient mutant data as gray bars, for 6 h at an MOI of 10, and supernatants were analyzed for (A) TNF and (B) IL-6. The data are displayed as means ± SEM from at least 3 experiments, as analyzed by unpaired *t* test.

The cytochalasin D data demonstrated that increased MDM inflammatory responses when infected with the TIGR4 strain compared to the TIGR4 Δ*cps* strain persisted even after phagocytosis was blocked. To assess whether the effects of the capsule on inflammatory responses were restricted to TIGR4, MDMs were incubated with the wild type and unencapsulated mutants for three further S. pneumoniae strains, including recent clinical capsular serotype 6B and 23F isolates. There were significantly higher levels of TNF and IL-6 in supernatants when MDMs were incubated with the wild type in comparison to unencapsulated bacteria for two serotypes (2 and 6B), but not for serotype 23F (Fig. 8A and B). These data show that encapsulated S. pneumoniae strains are more proinflammatory than unencapsulated strains for multiple (although not all) serotypes, and the role of the central TLR2 pathway in this effect was explored in more detail.

**FIG 9:**
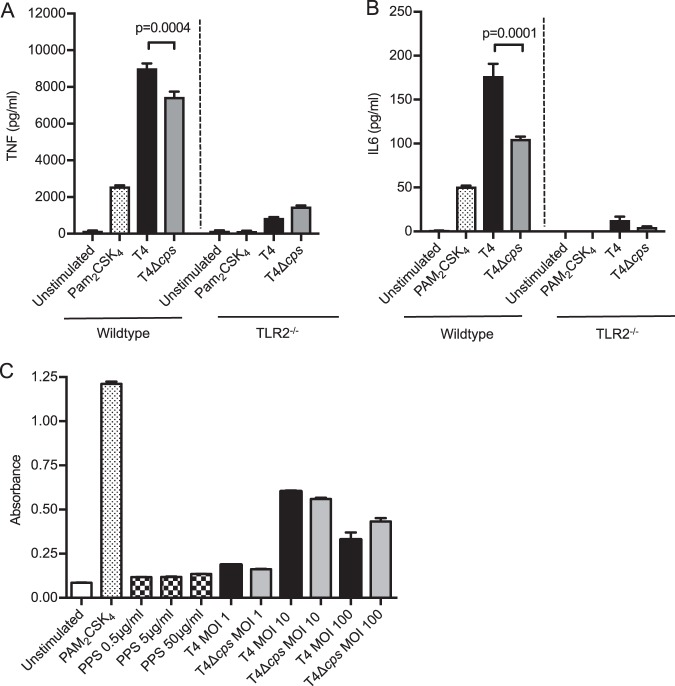
(A and B) BMDMs from wild-type or TLR2 knockout C57BL/6 mice were incubated with controls, WT TIGR4, or the TIGR4 Δ*cps* mutant at an MOI of 10 for 4 h. Levels of (A) TNF and (B) IL-6 were measured in supernatant and are displayed as means ± SEM from 3 experiments, as analyzed by unpaired *t* test. (C) HEK TLR2 cells were incubated with controls, PPS at various concentrations, or bacteria at various MOI for 16 h. Supernatant was analyzed for absorbance at 490 nm, and results are displayed as means ± SEM from 3 experiments, with WT TIGR4 data shown as black bars, TIGR4 Δ*cps* data as gray bars, and purified PPS data as hatched bars. Analysis was by 1-way ANOVA and Tukey’s multiple-comparison test.

Infection of murine bone marrow-derived macrophages (BMDMs) with TIGR4 strains demonstrated that macrophage TNF and IL-6 responses to S. pneumoniae were, as expected (17), highly dependent on TLR2. Although differences in the TNF responses between TIGR4 and TIGR4 Δ*cps* strains were lost after infection of TLR2^−/−^ macrophages (Fig. 9A and B), the TIGR4 and TIGR4 Δ*cps* strains both stimulated a TLR2 reporter assay to a similar degree and purified serotype 4 capsular polysaccharide (PPS) had no effect (Fig. 9C). These results indicate that although the macrophage cytokine response to S. pneumoniae is dependent on TLR2, the capsule does not directly affect TLR2 activation. Pneumolysin has multiple effects on immune responses to S. pneumoniae, including influencing inflammatory responses (20), and could be a confounding factor influencing the MDM inflammatory response. We therefore assessed pneumolysin activity in the TIGR4 Δ*cps* mutant strain by using a red cell lysis assay and a double-knockout Δ*ply* Δ*cps* strain as a control. These data showed no significant differences in red cell lysis between TIGR4 wild-type and TIGR4 Δ*cps* strains (Fig. S1C). Compatible with recent data showing that pneumolysin inhibits production of proinflammatory cytokines (38), there was increased production of IL-6 and TNF when MDMs were incubated with pneumolysin-deficient strains, with no differences seen between the wild-type and unencapsulated strains (Fig. S1D and E). These data show that the increased production of inflammatory cytokines in response to encapsulated strains compared to unencapsulated strains is unlikely to be related to the effects of pneumolysin.

## DISCUSSION

Multiple cell surface and intracellular signaling pathways have been identified that lead to an inflammatory response when triggered by bacterial interactions with macrophages (6, 8, 10, 20–22, 24, 39–41). The dominance of specific pathways in response to particular pathogens will influence disease pathogenesis, but there are few published data on the relative contributions of intra- and extracellular pathways for inflammatory responses to bacterial pathogens. Here, we have investigated the contribution of intracellular bacteria to the macrophage inflammatory response to S. pneumoniae using differences in susceptibilities to phagocytosis of encapsulated and unencapsulated S. pneumoniae. We have made two major perhaps unexpected observations: (i) the early macrophage proinflammatory cytokine response to S. pneumoniae is mainly driven by extracellular bacteria, with little effect of internalized bacteria, and (ii) for three of the four serotypes investigated, encapsulated bacteria are proinflammatory in comparison to their isogenic unencapsulated derivatives.

The dominant role of extracellular bacteria for the macrophage inflammatory response to S. pneumoniae was suggested by the reduced inflammatory responses to the unencapsulated strain despite the increased susceptibility of this strain to phagocytosis compared to encapsulated S. pneumoniae. These results were supported by data obtained after nonlytic killing of extracellular bacteria using gentamicin, inhibiting phagocytosis using cytochalasin D, and using opsonized bacteria. Both the transcriptomic and supernatant cytokine level data demonstrate that production of protective inflammatory cytokines by macrophages infected with S. pneumoniae was independent of phagocytosis and internalization. These results contrast strongly for data obtained from another extracellular Gram-positive pathogen, Staphylococcus aureus, for which TNF and other inflammatory cytokine responses depended on a combination of extracellular and intracellular TLR activation (25). In contrast to our data, when phagocytosis of S. aureus was blocked, there were reductions in macrophage cytokine responses, and a mutant S. aureus strain that was more sensitive to intraphagosome degradation caused increased inflammatory responses (25, 26). Given the similarities between S. aureus and S. pneumoniae in disease pathogenesis and the PRRs they are known to activate (42–44), the contrast between them in mechanisms driving macrophage inflammatory responses is surprising. Phagocytosis by innate immune cells also induced more inflammation for the spirochete Borrelia burgdorferi (45, 46). One difference in the biology of these pathogens that might be relevant is that the majority of phagocytosed S. pneumoniae cells are killed within 4 h (Fig. 1), whereas S. aureus is able to survive in significant numbers for at least 24 h (25, 26). Host cells scale their inflammatory response to match the degree of threat from the invading pathogen, which for S. pneumoniae could potentially lead to the reduced responses to internalized bacteria reflecting this pathogen’s relatively poor intracellular survival (47).

As the capsule blocks S. pneumoniae interactions with MDM phagocytic receptors, our initial hypothesis was that the presence of the capsule would reduce proinflammatory signaling by macrophages due to reduced internalization and inhibition of bacterial-host interactions with external proinflammatory PRRs such as TLR2, similar to the effect of the capsule for some other pathogens (48–52). However, unexpectedly, MDMs incubated with encapsulated S. pneumoniae secreted higher levels of TNF (and to a lesser extent IL-1β and IL-6) and had increased expression of a range of proinflammatory and chemokine genes, including TNF, IL-1β, IL-6, IL-23A, CXCL1, and CXL3. During culture with MDMs, encapsulated S. pneumoniae CFU increased over time compared to the unencapsulated strain, which probably reflects the increased susceptibility of the latter to phagocytosis. However, several strands of evidence support that the differences in the MDM inflammatory response between the encapsulated and unencapsulated strains were not purely related to differences in CFU. These included the following: (i) multiple MOI, which showed that increased TNF responses to encapsulated S. pneumoniae persisted when MDMs were incubated with 1/10 or 1/100 the number of TIGR4 bacteria compared to TIGR4 Δ*cps*; (ii) killing extracellular S. pneumoniae using gentamicin after 4 h of incubation, when there were no significant differences in CFU between the TIGR4 and TIGR4 Δ*cps* strains, still caused large differences in TNF responses, and (iii) repeating the experiments with three additional S. pneumoniae serotypes, two of which also showed increased inflammatory responses to the encapsulated strain compared to the unencapsulated mutant derivatives. In addition, an important observation is that only a small number of genes showed differences in transcriptional responses between the TIGR4 and TIGR4 Δ*cps* strains, and these were largely limited to inflammatory response genes, whereas broader changes across the whole MDM transcriptional response to S. pneumoniae might be expected if the differences were being driven by CFU alone. Increases in host transcriptional responses have also been described when encapsulated serotype 2 S. pneumoniae cells were incubated with human pharyngeal epithelial cells compared to the unencapsulated strain (53).

Our data using mouse infection models suggested the increased inflammatory responses to encapsulated bacteria could be relevant during disease. There was an increased early BALF TNF response after infection with the TIGR4 strain compared to infection with the TIGR4 Δ*cps* strains in a model of early pneumonia, with differences persisting when using different inoculum doses, nonreplicating bacteria, and at time points when TIGR4 and TIGR4 Δ*cps* strains showed no significant differences in BALF CFU (e.g., 4 h data after low-dose infection). However, the mouse data need to be interpreted cautiously as even when similar BALF CFU occur for the TIGR4 and TIGR4 Δ*cps* strains at a single time point, the different inoculum sizes needed to achieve this mean the mice would have been exposed to markedly different bacterial loads over the total duration of the infection.

These results suggest that as well as assisting immune evasion, the capsule also promotes a stronger inflammatory response, which mouse data suggest could potentially aid S. pneumoniae transmission (54). Although inflammation is essential for control of S. pneumoniae infections (4–7, 10, 11), this effect of the capsule in promoting inflammation could also exaggerate the severity of invasive infections by contributing to the development of ARDS or septic shock and leading to more tissue damage during pneumonia (55) and meningitis (56).

The mechanism by which encapsulated S. pneumoniae cells stimulate a stronger MDM inflammatory response remains unclear. We confirmed published data that the purified capsule has minimal effect on inflammatory cytokine release (33–35), with residual responses after inhibition of LPS responses only to very high nonphysiological concentrations of capsular material. Furthermore, expression of the S. pneumoniae serotype 4 capsule did not increase the inflammatory response to S. mitis. In a reporter assay, capsule material did not stimulate a TLR2 response, and encapsulated and unencapsulated S. pneumoniae cells were able to cause similar levels of TLR2 stimulation. One possible explanation is that the increase in intracellular bacteria for the unencapsulated strains inhibited inflammatory cytokine responses to S. pneumoniae, but preventing phagocytosis did not reduce the differences between TIGR4 and TIGR4 Δ*cps* in inflammatory responses at qPCR, whole-genome transcriptional, and secreted protein levels.

In summary, we have shown that in contrast to S. aureus, macrophage-dependent inflammatory responses to S. pneumoniae capsule are largely dependent on extracellular bacteria. In addition, we have shown an unexpected proinflammatory effect on macrophage responses of incubation with encapsulated compared to unencapsulated S. pneumoniae. Further investigation will be necessary to identify why the early MDM inflammatory response to S. pneumoniae infection is largely independent of intracellular signaling pathways and is stronger in response to encapsulated compared to unencapsulated bacteria.

## MATERIALS AND METHODS

### Bacterial strains and culture.

Experiments were performed using the wild-type serotype 4 TIGR4 S. pneumoniae strain and its otherwise isogenic unencapsulated mutant derivative, TIGR4 Δ*cps* (a kind gift from J. Weiser) (57). Δ*pabB* mutants (58), which do not replicate without an exogenous source of *para*-aminobenzoic acid, were used to address differences in bacterial numbers *in vivo*. S. pneumoniae cells were cultured in THY broth (Todd-Hewitt broth supplemented with yeast extract) or on 5% blood Columbia agar plates (Sigma-Aldrich, Gillingham) at 37°C in 5%CO_2_. S. pneumoniae cells were labeled fluorescently for uptake experiments using 6-carboxyfluorescein succinimidyl ester (FAM-SE [Molecular Probes, Eugene, OR, USA]) as previously described (59). The S. mitis wild-type strain, S. mitis Δ*cps* mutant, and an S. mitis mutant strain expressing the S. pneumoniae serotype 4 capsule were used to address the effect of capsule in a nonpathogenic species (37). Additional experiments used encapsulated and unencapsulated strains (made by deletion of *cps* Janus cassette as previously described [57] or of *cpsD* for the serotype 2 strain [60]) of serotypes 2 (D39), 6B (ST138 clinical isolate), and 23F (ST36 clinical isolate). A TIGR4 Δ*cps* Δ*ply* strain was created by using DNA from TIGR4 Δ*cps* and TIGR4 Δ*ply* (a kind gift from Tim Mitchell) strains, using antibiotic resistance cassettes to allow selection of double-knockout mutants (61).

### Cell culture.

Blood from healthy volunteers was layered onto Ficoll-Paque Plus (GE Healthcare Life Sciences, Hatfield, United Kingdom) and centrifuged to obtain peripheral blood mononuclear cells (PBMCs). Adherent cells were differentiated in Roswell Park Memorial Institute medium (RPMI), supplemented with human macrophage colony-stimulating factor (M-CSF) and autologous serum, into monocyte-derived macrophages (MDMs) as previously described (62). Experiments were carried out in RPMI supplemented with 5% pooled human AB serum (Sigma-Aldrich, Gillingham, United Kingdom). Experiments using MDMs were approved by the joint University College London/University College Hospitals National Health Service Trust Human Research Ethics Committee (reference no. 3076/001). RAW 264.7 cells, a murine cell line with a macrophage-like phenotype, were cultured in RPMI (Gibco, Loughborough, United Kingdom) supplemented with fetal bovine serum (FBS [Lonza, Blackley, United Kingdom]) and l-glutamine. HEK 293 cells, a human embryonic kidney cell line, stably transfected with TLR2 and CD14 (Invivogen, San Diego, CA) were cultured in Dulbecco’s modified Eagle’s medium (DMEM [Gibco, Loughborough, United Kingdom]) supplemented with FBS and l-glutamine. THP-1 cells, a human monocyte cell line, were cultured in RPMI and FBS with l-glutamine. Bone marrow was extracted from 6- to 8-week-old C57BL/6 WT and *TLR2*^−/−^ mice (Jackson ImmunoResearch Laboratories) and differentiated into bone marrow-derived macrophages (BMDMs) for 7 days in L929-conditioned medium using standard protocols (63).

### Red cell lysis assay.

Two percent horse blood was added to U-bottom 96-well plates with serial dilutions of bacteria or 0.5% saponin (positive control) in phosphate-buffered saline (PBS). The plates were incubated at 37°C in 5% CO_2_ for 30 min and then centrifuged. The supernatant was aspirated and placed in a flat-bottom 96-well plate (Brand, Wertheim, Germany), and absorbance at 540 nm was measured on a microplate reader. Absorbance reflected free hemoglobin liberated from lysed cells (64).

### Phagocytosis assays.

Antibiotic protection assays were carried out using 200 μg/ml gentamicin as previously described (65). In brief, this involved incubating MDM or RAW cells with bacteria with or without antibiotics, washing the cells after 2 h, and then plating the lysate for bacterial counts to measure internal bacteria versus adherent plus internal bacteria. Intracellular survival experiments were performed using THP-1 cells, with MOI resulting in approximately equal uptake per THP-1 cell of TIGR4 or TIGR4 Δ*cps* bacteria, followed by addition of gentamicin (200 μg/ml) after 30 min of incubation to kill extracellular bacteria and then addition of 2% saponin at the specified time points to lyse the cells for plating to obtain bacterial CFU. Microscopy phagocytosis assays were performed using FAM-SE-labeled bacteria opsonized in human serum, staining of external bacteria with phycoerythrin (PE)-labeled goat anti-human IgG (Sigma-Aldrich, Gillingham), and DAPI (4′,6-diamidino-2-phenylindole) staining of the nuclei, using a Hermes microscope for imaging (Biotech-Europe, Prague, Czech Republic). Images were analyzed on Metamorph (Metamorph, Inc.) software. Flow cytometry phagocytosis assays were performed using FAM-SE-labeled S. pneumoniae as previously described (59).

### TLR2 reporter assay.

HEK TLR2 cells were resuspended in HEK-Blue detection medium (Invivogen, San Diego, CA), added to 96-well tissue culture plates (Nunc, Roskilde, Denmark), and then stimulant was added. The optical density, reflecting TLR2 activation, was measured on a microplate reader (Versamax, Sunnyvale, CA, USA).

### Cytokine ELISA.

Enzyme-linked immunosorbent assays (ELISAs) were carried out using R&D Systems (Abingdon) Duo kits as per the manufacturer’s instructions.

### qPCR.

RNA extraction of MDM for qPCR or microarray was performed with Qiagen RNeasy minikit (Qiagen, Hilden, Germany) as per the manufacturer’s instructions. Contaminating DNA was eliminated with Precision DNase (Primer Design, Southampton, United Kingdom). RNA concentration and quality was measured using a Nanodrop 3000 and a ratio of absorbance of 260:280 (aiming for 2.0). cDNA was synthesized in clear PCR plates (Thermoscientific, Loughborough, United Kingdom) with qScript cDNA SuperMix (QuantaBiosciences, Beverley, MA, USA). TaqMan gene expression assays were run on a Realplex Mastercycler (Eppendorf, Stevenage, United Kingdom). The cycle threshold (*C_T_*) was determined for all samples and analyzed by Δ*C_T_* for relative expression values, with the GAPDH (glyceraldehyde-3-phosphate dehydrogenase) gene used as a housekeeping gene (66).

### Microarray whole-genome transcription.

Total RNA was purified from MDM lysates, collected as for qPCR, and processed for Agilent microarrays as previously described (66, 67).

A microarray (Agilent, Santa Clara, CA, USA) platform was used to perform whole-genome transcriptional analysis of MDMs incubated with bacteria. An Agilent Low-input Quick Amp labeling kit was used for two-color microarray-based gene analysis on an Agilent microarray scanner. Data from the microarray were normalized as previously described (70) and analyzed using a multiexperiment viewer (Sourceforge) and Bioagilent insert for R (R Project). Bioinformatic analysis was performed using the online analysis tools Innate DB (68), Gephi (v0.82) for network visualization (https://gephi.org/), and oPOSSUM (69).

### Mouse infection model.

Murine work was carried out within Home Office guidelines under project license PPL70/7361. Female CD1 5-week-old mice (Charles Rivers) were anesthetized with isoflurane and inoculated intranasally with 50 μl of bacterial suspension as previously described (17, 32). In specific experiments, antibodies or inhibitors were administered intranasally under isoflurane anesthesia or intraperitoneally. At specified time points, the mice were euthanized with intraperitoneal pentobarbital, and bronchoalveolar lavage fluid (BALF), blood, and lungs (homogenized using cell strainers [Falcon, Corning, NY, USA]) were obtained to calculate bacterial CFU by plating serial dilutions. BALF cell counts were counted using a hemocytometer.

### Statistical analysis.

Statistical analyses of data were performed with GraphPad Prism V7 (La Jolla, CA, USA). Data were analyzed by unpaired *t* test when comparing 2 groups and 1- or 2-way analysis of variance (ANOVA) with Tukey’s or Sidak’s multiple-comparison test, respectively, for multiple groups. Nonparametric data (e.g., CFU) were analyzed by Mann-Whitney U test when comparing 2 groups, and Kruskal-Wallis with Dunn’s multiple-comparison test for multiple groups. Statistically significant differences in microarray data were identified by unpaired *t* test. Z score analysis was performed on transcription factor binding bioinformatics.

### Data availability.

All microarray data used in this study are available in ArrayExpress (https://www.ebi.ac.uk/arrayexpress/) under accession no. E-MTAB-5894.
