# Supplementary figures and images for: Relative Contributions of Extracellular and Internalized Bacteria to Early Macrophage Proinflammatory Responses to Streptococcus pneumoniae
Source: mBio. 2019 Sep 24;10(5):e02144-19. doi: 10.1128/mBio.02144-19 (PMC6759765; doi:10.1128/mBio.02144-19)

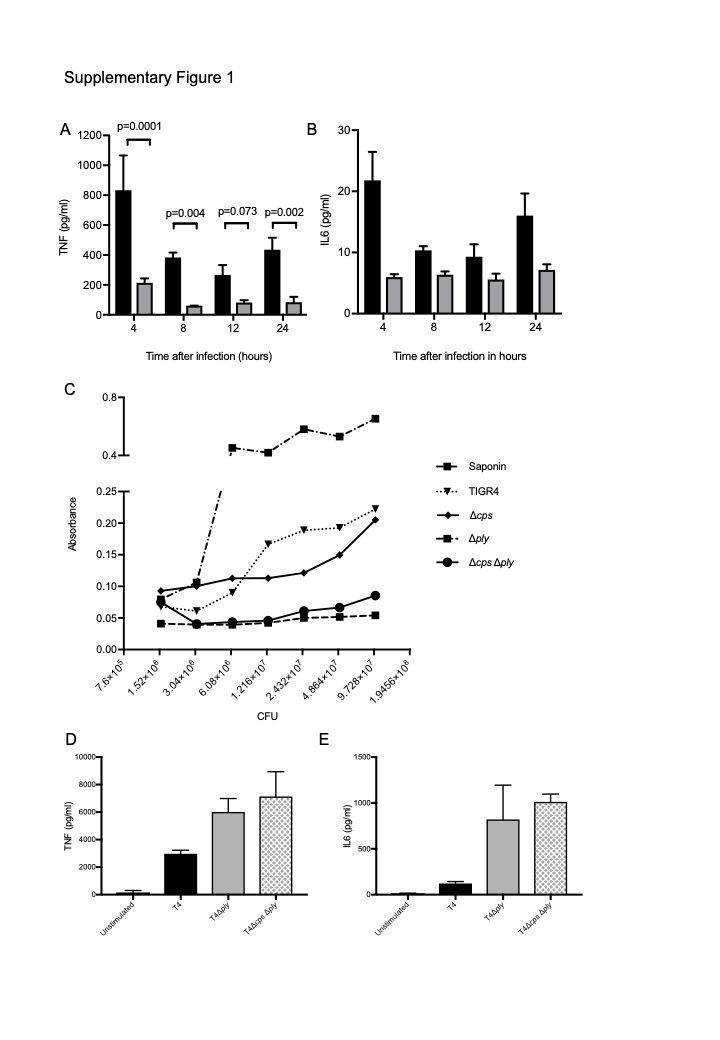

Supplement: FIG S1 [file mBio.02144-19-sf001.tif]
